# Gating of β-Barrel Protein Pores, Porins, and Channels: An Old Problem with New Facets

**DOI:** 10.3390/ijms241512095

**Published:** 2023-07-28

**Authors:** Lauren A. Mayse, Liviu Movileanu

**Affiliations:** 1 Department of Physics, Syracuse University, 201 Physics Building, Syracuse, NY 13244, USA; lamayse@syr.edu; 2 Department of Biomedical and Chemical Engineering, Syracuse University, 223 Link Hall, Syracuse, NY 13244, USA; 3 The BioInspired Institute, Syracuse University, Syracuse, NY 13244, USA

**Keywords:** membrane proteins, electrophysiology, protein folding, single-molecule dynamics, conformational transitions

## Abstract

β barrels are ubiquitous proteins in the outer membranes of mitochondria, chloroplasts, and Gram-negative bacteria. These transmembrane proteins (TMPs) execute a wide variety of tasks. For example, they can serve as transporters, receptors, membrane-bound enzymes, as well as adhesion, structural, and signaling elements. In addition, multimeric β barrels are common structural scaffolds among many pore-forming toxins. Significant progress has been made in understanding the functional, structural, biochemical, and biophysical features of these robust and versatile proteins. One frequently encountered fundamental trait of all β barrels is their voltage-dependent gating. This process consists of reversible or permanent conformational transitions between a large-conductance, highly permeable open state and a low-conductance, solute-restrictive closed state. Several intrinsic molecular mechanisms and environmental factors modulate this universal property of β barrels. This review article outlines the typical signatures of voltage-dependent gating. Moreover, we discuss recent developments leading to a better qualitative understanding of the closure dynamics of these TMPs.

## 1. The Structure and Composition of β Barrels

Cellular and subcellular membranes include transmembrane proteins (TMPs) that facilitate solute transport and signaling. The first class of TMPs encompasses transmembrane α helices. These hydrophobic proteins are the most abundant among both prokaryotes and eukaryotes. The second class of TMPs includes β barrels, which are folded protein scaffolds made of anti-parallel β strands. A fundamental property of α-helical transmembrane proteins is that they feature continuous hydrophobic stretches of residues across the lipid membrane. In contrast, β barrels are made of polypeptide chains with alternating hydrophobic and hydrophilic residues. This way, a β-barrel structure is like a cylinder with an external hydrophobic interface oriented toward the lipid membrane and an internal hydrophilic surface surrounding an aqueous transmembrane channel. Hence, these TMPs may serve as conduits for transporting nutrients and small-molecule metabolites across membranes. Remarkably, the β strands are connected through a network of numerous hydrogen bonds between the amide and carbonyl groups of the polypeptide backbone. This dense hydrogen bonding distribution is the fundamental molecular mechanism by which a β-barrel scaffold attains its unusually high mechanical and thermodynamic stability [1]. In addition, β barrels have aromatic side chains at the water–membrane interface, thus forming stabilizing contacts with the polar headgroups and hydrophobic tails of surrounding lipids. Such TMPs are present in the outer membranes (OM) of mitochondria, chloroplasts, and Gram-negative bacteria [2,3,4,5,6,7,8]. Moreover, β-barrel structures are also formed by various pore-forming toxins (PFTs) [9,10,11,12].

**Figure 1:**
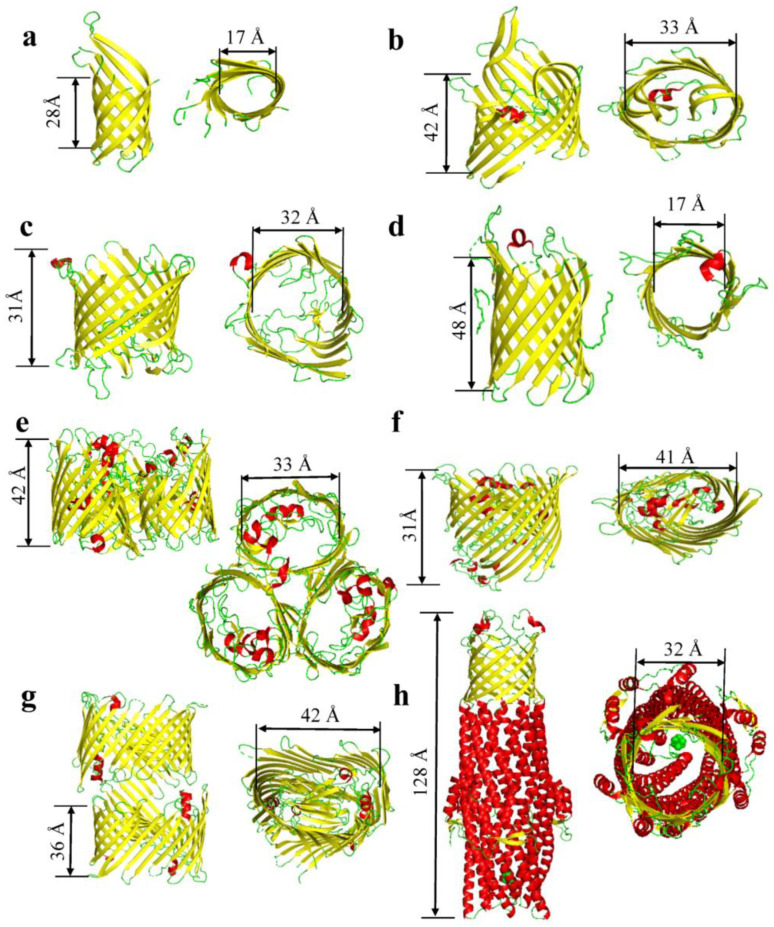
β-barrel proteins of Gram-negative bacteria. (**a**) OmpA (PDB:1QJP; [15]). (**b**) OmpT (PDB:6EHD; [39]). (**c**) OprD (OccD1) (PDB:3SY7; [40]). (**d**) OmpG(PDB:2F1C; [31]). (**e**) OmpF(PDB:2ZFG; [41]). (**f**) FhuA(PDB:1BY3; [16,17]). (**g**) PapC(PDB:3FIP; [18]). (**h**) TolC(PDB:7NG9; [42]).

In Gram-negative bacteria, the β strands are connected by short β turns (e.g., 4–6 residues in length) [13] on the periplasmic side and long flexible loops on the extracellular side. The size, flexibility, and conformation of various loops vary significantly in OM β-barrel proteins. They may have an important functional role, providing specificity to individual barrels. The loops can be oriented toward the extracellular side, or they can fold back into the pore lumen, drastically reducing the cross-sectional internal diameter of the hydrophilic channel. Hence, they can regulate small-molecule permeability and selectivity. Most OM proteins from Gram-negative bacteria form a β-barrel structure comprising 8–24 β strands [6,14] (Figure 1; Table 1). Many β barrels exist as monomers (e.g., OmpA [15] and FhuA [16,17]). Yet, they can also oligomerize in various ways, to generate either multimeric structures of distinct β barrels (e.g., dimeric PapC [18] or trimeric OmpF [19,20] and OmpC [21]) or a single β barrel made of a few polypeptide chains (e.g., the trimeric TolC of *Escherichia coli* [22,23]).

The narrowest monomeric OM β-barrel proteins formed by eight β strands include OmpA [15], OmpW [24,25], OmpX [26], and PagP [27]. OmpA [28,29], an essential virulence factor facilitating eukaryotic cell infection and antibiotic resistance, represents the most abundant OM β-barrel protein in *E. coli*. The monomeric OM proteins OmpT [30] and OmpG [31,32,33] of *E. coli* contain 10 and 14 β strands, respectively. The monomeric β-barrel protein ferric hydroxamate uptake component A (FhuA) [16,17] and the lipopolysaccharide (LPS) channel (LptD) [34] encompass 22 and 26 β strands, respectively. SprA, a protein-conducting translocon of the type 9 secretion system (T9SS), is a 36-stranded OM protein [35]. This is the widest single-polypeptide β-barrel known to date. The OM β-barrel proteins from Gram-negative bacteria execute various tasks, such as specific porins (e.g., OprD [36] and OpdK [37] of *Pseudomonas aeruginosa*), passive-diffusion porins (e.g., OmpF [19,20] and OmpC [21]), enzymatic elements (e.g., the protease OmpT [30], lipase OMPLA [38], and acyltransferase PagP [27]), adhesin (e.g., OmpX [26]) and structural (e.g., OmpA [15,28,29]) proteins, secretion pathways (e.g., PapC [18]), efflux channels and pumps (e.g., TolC [22,23]), and active transporters (e.g., FhuA [16,17]).

**Figure 2:**
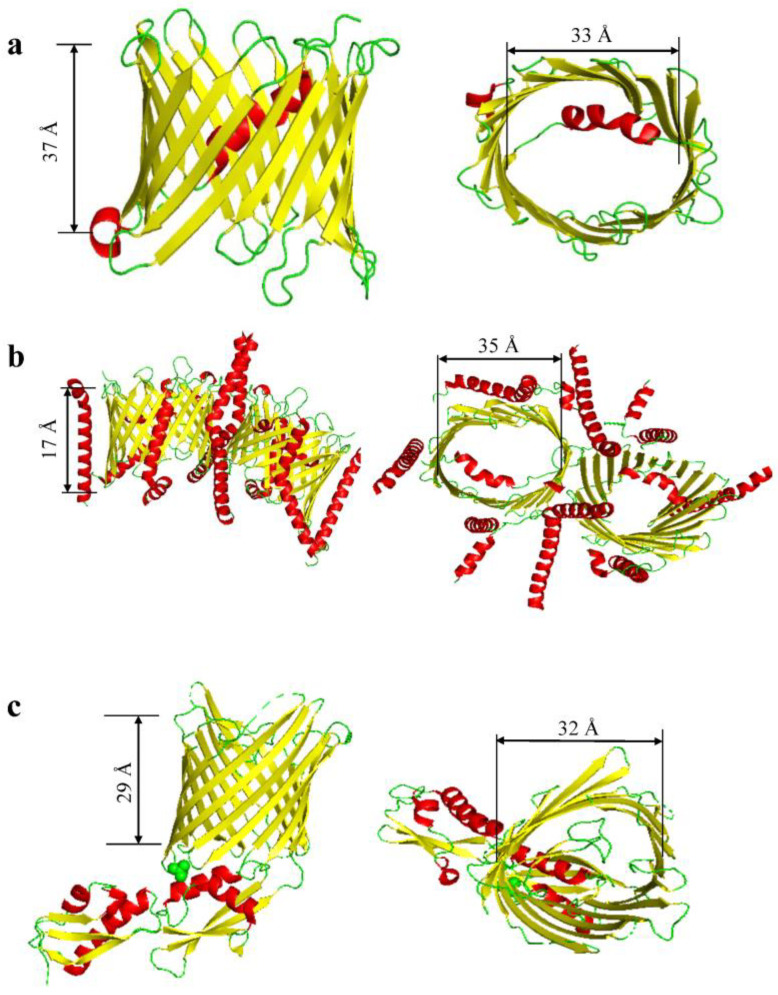
Mitochondrial β-barrel proteins. (**a**) VDAC-1 (porin) from *H. sapiens* (PDB:6TIQ; [43]). (**b**) TOM complex from *H. sapiens* (PDB:7VD2; [44]). (**c**) FhaC from *E. coli* (PDB:4QKY; [45,46,47]).

**Figure 3:**
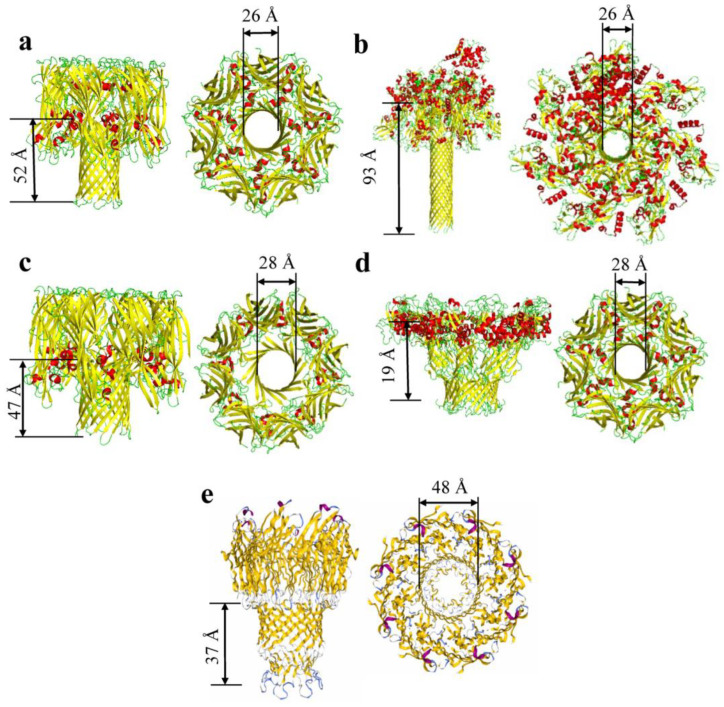
β-barrel pore-forming toxins. (**a**) α-hemolysin of *S. aureus* (PDB:4ANZ; [48,49,50]). (**b**) Anthrax toxin with lethal factor side and top view (PDB:6PSN; [51]). (**c**) γ–hemolysin from *S. aureus* (PDB:3B07; [9]). (**d**) Aerolysin prepore side and top view from *A. hydrophila* (PDB: 5JZH/5JZW; [12]). (**e**) MspA of *M. smegmatis* (PDB:1UUN; [52]).

Below, Figure 1, Figure 2 and Figure 3 and Table 1, Table 2 and Table 3 are presented.

**Table 1:** β-barrel proteins of outer membranes of Gram-negative bacteria. Molecular weights were determined using the UniProt profile from each protein database file. The PBD code for each protein is included. These codes were used to determine the oligomeric state. The average internal diameters do not include the side chains of the internal residues. Hence, these diameters are calculated from C_α_ to C_α_ atoms. The number of strands, average diameter, length of barrel, corks, and loops were all determined using the PyMOL Molecular Graphics System (Version 2.4.0; Schrödinger, LLC, New York, NY, USA).

Proteins	PDB Code	Average Molecular Weight(kDa)	Oligomeric State	Number of β-Strands per Monomer	Average Internal Diameter (Å)	Length of Barrel (Å)	Corks and Loops	Citation
OmpA	1QJP	37	Mono/Dimer	8	17	28	4 Loops	[15]
OmpW	2F1V/2F1T	21	Monomer	8	17	23	4 Loops	[24,53]
OprD	3SY7	48.4	Monomer	8	35	34	4 loops	[40]
OmpT	6EHD	40	Trimeric	10	33	42	8 Loops	[39]
OmpG	2F1C	35	Monomer	14	17	48	7 loops	[31]
OmpF	2ZFG	40	Trimer	16	33	42	8 Loops	[41]
OmpC	2J1N	40.4	Trimer	16	32	35	8 loops	[21]
PhoE	1PHO	39.5	Trimer	16	32	39	8 loops	[54]
Maltoporin	1AF6	49.9	Trimer	18	37	35	9 loops	[55]
FhuA	1BY3	82	Monomer	22	41	31	1 Cork & 11 loops	[17]
PapC	3FIP	91.5	Dimer	24	42	36	1 cork & 12 loops	[18]
TolC	7NG9	162	Trimer	6	32	128	6 loops	[42]

**Table 2:** Mitochondrial β-barrel proteins. Molecular weights were determined using the UniProt profile from each protein database file. The PBD code for each protein is included. These codes were used to determine the oligomeric state. The average internal diameters do not include the side chains of the internal residues. Hence, these diameters are calculated from C_α_ to C_α_ atoms. The number of strands, average diameter, length of barrel, and loops were all determined using the PyMOL Molecular Graphics System (Version 2.4.0; Schrödinger, LLC).

Proteins	PDB Code	Average Molecular Weight(kDa)	Oligomeric State	Number of β-Strands per Monomer	Average Internal Diameter (Å)	Length of Barrel (Å)	Corks and Loops	Citation
VDAC	6TIQ	31	Dynamic (Dimer, Trimer, Tetramer)	19	33	37	9 loops	[43]
TOM complex	7VD2	38	Dimer	19	35	17	9 loops	[44]
Fhac/Sam50	4QKY	54.4	Hexamer	16	32	29	8 loops	[45,46,47]

**Table 3:** β-barrel pore-forming toxins. Molecular weights were determined using the UniProt profile from each protein database file. The PBD code for each protein is included. These codes were used to determine the oligomeric state. The average internal diameters do not include the side chains of the internal residues. Hence, these diameters are calculated from C_α_ to C_α_ atoms. The number of strands, average diameter, and length of barrel were all determined using the PyMOL Molecular Graphics System (Version 2.4.0; Schrödinger, LLC).

Toxins	PDB	Average Molecular Weight (kDa)	Number of Chains	Internal Diameter (Å)	Length of Barrel (Å)	Number of β-Strands	Citation
Cytolysin (Sticholysin II)	1GWY	19.3	1	14	23	10	[56]
α-hemolysin	3ANZ	33	7	26	52	14	[48,49,50]
γ-hemolysin	3B07	36.7	8	28	47	16	[9]
Bi-component Toxin LukGH	4TW1	36.8	8	30	39	16	[57]
Aerolysin	5JZH/5JZW	54.3	7	28	19	14	[12]
Epsilon toxin	6RB9	36.3	7	30	68	14	[58]
Anthrax Toxin	6PSN	90	7	26	93	14	[51]
Lysenin	5GAQ	33.4	9	34	85	18	[59]
MspA	1UUN	22.1	8	48	37	16	[52]

In the OMs of human mitochondria, the most abundant β barrels are the 16-stranded Sam50 [45,46], the 19-stranded voltage-dependent anion-selective channel isoform 1 (VDAC1) [60], and the translocase complex of the OM (TOM) [44] (Figure 2; Table 2). It should be noted that both VDAC1 and TOM are structurally distinctive from bacterial OM β barrels because of their odd number of strands. VDAC barrels include three isoforms that result from evolutionary processes and distinct adaptations to a diverse subset of functional roles and interactomes [61,62,63,64,65,66]. The mammalian β barrels feature various functionalities in cellular signaling and apoptosis.

The OM of chloroplasts also contains various β-barrel proteins such as the outer envelope proteins 37 (OEP37 [67,68]). They function as transporters for small-molecule nutrients, peptides, and nucleic acids. Moreover, the OM includes the translocase of the outer chloroplasts envelope (Toc75) and the outer envelope protein 80 kD (OEP80), which have a focal role in the protein import into plastids [69,70].

Furthermore, many PFTs form β-barrel structures made from several protomers that assemble at the surface of a membrane for oligomerization and pore formation. The archetype of a homomeric β-barrel PFTs (β-PFTs) is the staphylococcal α-hemolysin, a heptameric protein of a known crystal structure [48]. This complex forms a mushroom-shaped assembly, and each protomer participates with two anti-parallel β strands to form a 14-stranded protein pore (Figure 3; Table 3). The protective antigen channel (PA_63_) of the anthrax toxin secreted by *Bacillus anthracis* is also a heptameric 14-stranded β barrel that facilitates the translocation of the edema factor and lethal factor proteins into the target cells [71]. In addition, β-PFTs can be formed by heteromeric complexes, such as bi-component toxins (e.g., leukocidins, γ-hemolysins, and Pantom-Valentine leukocidins (PVL)) [72,73,74]. Heteromeric β-PFTs require an interaction between the two distinct protomers [9,75,76]. In the past decade, new noncanonical β-barrel structures have been discovered for various transmembrane secretory systems. They include multimeric complexes made of a vast number of β strands mediating diverse protein secretion systems (e.g., CsgG [77] and secretins [78,79]). Another class of noncanonical β barrels is formed by cholesterol-dependent cytolysins that contain tens of protomers that co-participate in creating giant transmembrane protein pores with sizes between 50 and 200 antiparallel β strands [11].

## 2. Early Observations of Voltage Gating of β Barrels

Voltage gating is a biophysical process that implies the transient or permanent closure of a β-barrel protein pore, porin, or channel due to a transmembrane potential. As a result of this closure, the ionic flux is at least partly restricted. Early observations of voltage-dependent gating of various β barrels have been published by several groups, such as those of Rosenbusch [80,81], Lakey [82,83], Engelhardt [84,85], and Delcour [86,87,88,89]. An extensive amount of this pioneering work has been based on trimeric OM proteins OmpF [80,81,82,83,88,89,90,91,92,93], OmpC [86,87,94,95], and PhoE [81,82,88,96]. Single-molecule electrophysiology studies have reported voltage gating of porins under a broad range of experimental contexts that varied pH [97], salt concentration [98,99], membrane composition [100,101], method of channel reconstitution [89], electrostatic potential [102], and others. Different investigators observed diverse sensitivities to voltage gating in these early explorations. Yet, they agreed that different mechanisms mediate voltage gating. In addition, numerous native and mutated forms of β-barrel porins have been examined using single-channel electrical recordings [86,87,94] and full-atomistic molecular dynamics (MD) simulations [103,104,105,106,107,108,109] for determining biophysical properties such as ion permeation, unitary conductance, ionic selectivity, as well as the kinetics and dynamics of current gating fluctuations [110,111,112,113]. Several mechanisms of voltage-dependent gating were proposed, including the presence of charged residues within the constricted region of the pore interior and the motions of the long extracellular loops folding back into the pore lumen (e.g., L3 in OmpF). In addition, it was suggested that voltage gating occurs due to other intrinsic processes that correlate with the mechanical stability of β barrels. Later, it was identified that electrostatic effects [99,114,115] and pH [116,117] also play critical roles in the dynamics of current fluctuations and the overall stability of β barrels. In the following sections of this review article, we discuss the intrinsic spontaneous gating fluctuations of these TMPs and their regulatory mechanisms by specific environmental conditions.

## 3. Gating Activity Produced by Loops and Plugs

Many β-barrel OM proteins exhibit steric restrictions for ionic flow, such as long extracellular loops that fold back into the pore lumen and internal plug domains. In addition, their conformational moieties may cause reversible current gating in these TMPs [118,119]. For example, in *Pseudomonas aeruginosa*, substrate-specific 18-stranded porins have large external loops L3, L4, and L7 that partition into the pore lumen [120], producing a very narrow pore eyelet. Hence, these β barrels exhibit a relatively low-conductance open-substate current decorated by frequent fluctuations [37,40,120,121,122]. Interestingly, deletions of the internal loops L3 in OprD (OccD1) [36] and L7 in OpdK (OccK1) [123] increased in their single-channel conductance. These findings suggest that these loops directly participate in the constricted regions of these porins. Moreover, significant progress has been made in understanding the gating activities of monomeric OM proteins because of their potential in biotechnology. The primary benefit of these barrels is the opportunity to redesign them as single-polypeptide chain protein nanopores for single-molecule stochastic sensing. Notably, such a strategy would circumvent the necessity for separating the desired protein pore from other products of the assembly reaction; otherwise, a tedious sample preparation is required for multimeric protein pores [4,124,125,126,127].

**Figure 4:**
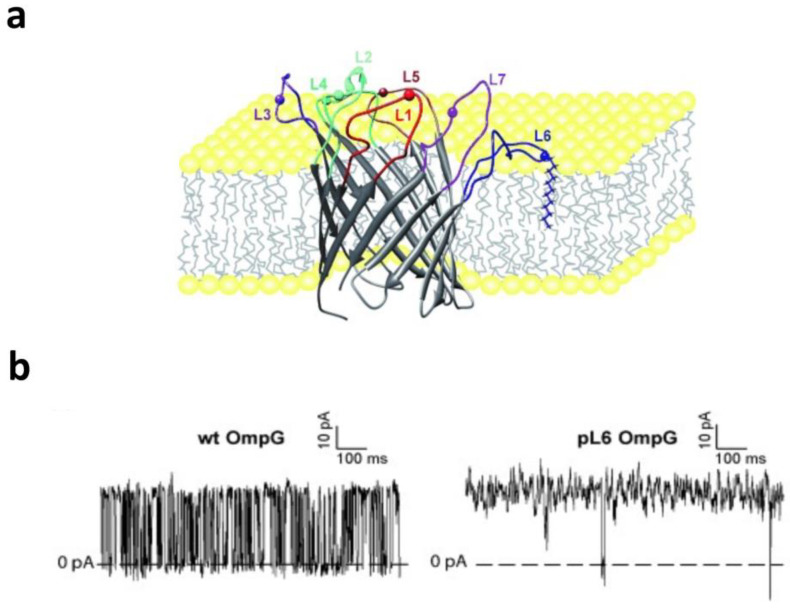
Loop 6 is crucial for the gating dynamics of OmpG. (**a**) This is a cartoon representation of loop L6 of OmpG being anchored into the lipid bilayer via dodecylation at Cys226. (**b**) Representative single-channel electrical recordings using the wild-type OmpG (**left** panel) and an OmpG mutant with the loop L6 immobilized onto the lipid bilayer, as shown in (**a**) (**right** panel). This figure was adapted from Zhuang and Tamm (2014) [128].

**Figure 5:**
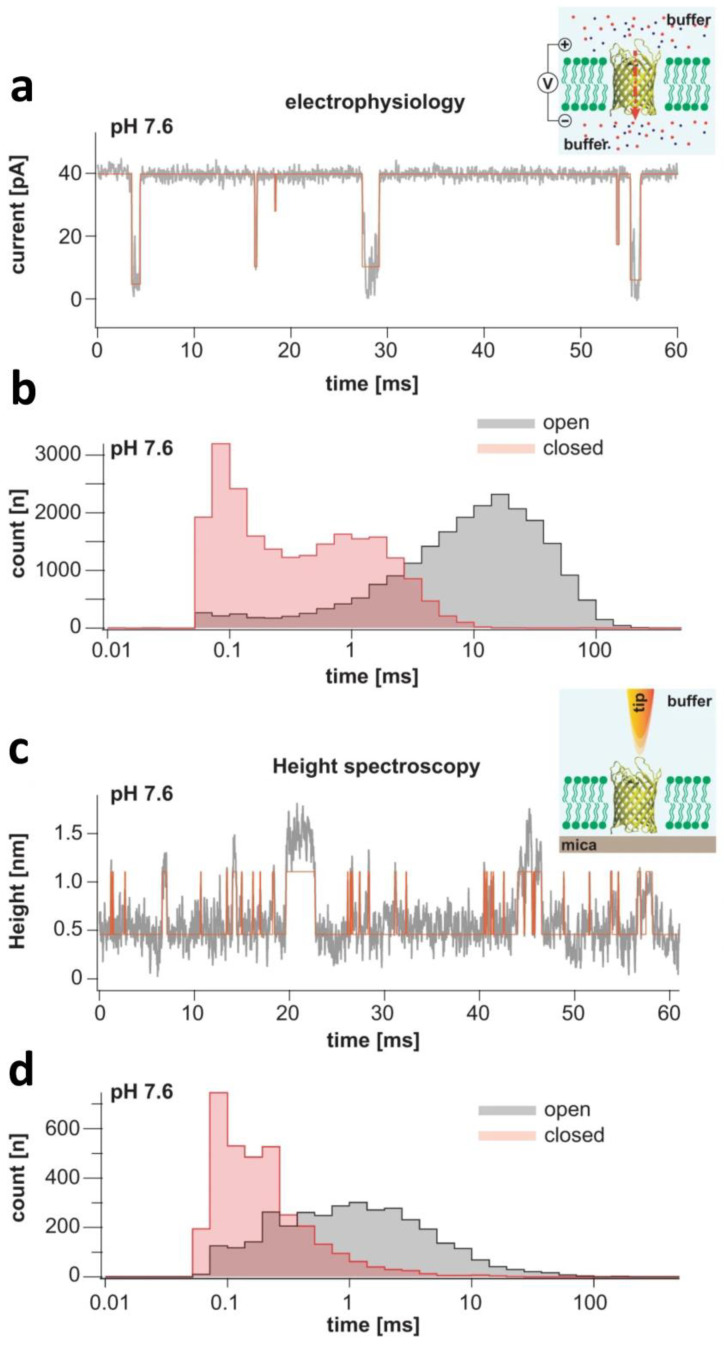
Gating evaluations of OmpG using single-molecule electrophysiology and high-speed AFM height spectroscopy (HS-AFM-HS). (**a**) A representative single-channel electrical trace of OmpG acquired at a transmembrane potential of +40 mV and pH 7.6. The schematic on the right side provides a scheme of the single-channel electrical recording experimental formulation. OmpG (yellow) is functionally reconstituted into a lipid bilayer (green). Potassium and chloride ions are indicated as red and blue spheres, respectively. The red arrow shows the direction of the ionic flow of cations at a positive applied potential. (**b**) A semilogarithmic dwell time histogram of the open and closed states, as determined by single-molecule electrophysiology. (**c**) A representative 60 ms long HS-AFM-HS recording that probes an OmpG protein functionally reconstituted into a lipid bilayer, which was suspended on mica at pH 7.6. The schematic on the right side is the HS-AFM-HS experimental setup. An AFM tip monitors conformational fluctuations of loop L6. (**d**) A semilogarithmic dwell time histogram of the open and closed states, as determined by HS-AFM-HS. Here, the low state indicates the open state, where the tip navigates within the pore lumen. The high state corresponds to the closed state, precluding the partitioning of the tip into the pore lumen. This figure was adapted from Sanganna Gari and coworkers (2021) [129].

Below, Figure 4 and Figure 5 are presented.

In the first example, OmpG [31,32,33,130], a monomeric 14-stranded β barrel that comprises seven extracellular loops, undergoes frequent current fluctuations around the open substate at neutral pH and an applied potential lower than 100 mV [112,113]. Protein engineering and MD simulations were utilized to produce a mutated OmpG with a 95% reduction in the gating activity [131,132]. This approach was achieved by the decrease in the moieties of the gating loop L6 [133] via (i) an exogenous disulfide bond engineered between strands β12 and β13, and (ii) the optimization of the β11-β12 inter-strand hydrogen bonding via an aspartic acid deletion. Thus, OmpG was the first monomeric β barrel engineered for acquiring a quiet open-substate for biosensing applications. These pioneering studies on the engineering of OmpG for biotechnological applications stimulated further developments for revealing mechanistic information about its gating activity. In a follow-up study, Zhuang and colleagues (2013) [128] used an innovative strategy for pinning individual extracellular loops of OmpG into the lipid bilayer (Figure 4a). This process has been conducted using the chemical modification of individual loops by long-hydrocarbon chain alkylation. This way, different OmpGs with one of the loops immobilized onto the lipid bilayer were systematically studied using NMR in detergent micelles and single-channel electrical recordings. Pinning loop L6 resulted in an OmpG protein pore with a highly reduced gating activity (Figure 4b). This discovery was in accordance with previous investigations that indicated the pivotal role of loop L6 in the voltage-gating function of OmpG [116,117,133]. Furthermore, this study provided key information about the structural and dynamic alterations of the neighboring and distant loops when one is immobilized onto the lipid bilayer. It also identified that in addition to loop L6, other extracellular loops contribute to channel closing and in different extents of cooperativity with L6.

In an independently conducted study by Grosse and coworkers [134], two L6 deletion variants of OmpG showed a unitary conductance like the wild-type OmpG but significant reductions in the gating activity. Intriguingly, a large truncation mutant of OmpG that encompassed deletions of all loops still exhibited a fivefold decrease in the gating activity with respect to the native protein. Therefore, a residual voltage gating of OmpG was independent of loop L6 conformational changes within the pore lumen. This gating activity may be determined by global changes in barrel conformation, resulting in the transient reduction in transmembrane ionic flux. These reversible structural fluctuations of the porin might involve its barrel stretching and compression [135]. For example, extensive breathing motions in VDAC1 (VDAC1), a 19-stranded β barrel, were determined using MD simulations and solid-state NMR spectroscopy (see below) [136].

Recently, Sanganna Gari and colleagues (2021) used a high-resolution AFM-based spectroscopy approach to provide time-resolved conformational fluctuations of loop L6 within the pore lumen of OmpG [129]. This method, also called high-speed AFM high spectroscopy (HS-AFM-HS), was utilized to find correlations of the physical conformational dynamics of the sample in the vertical direction with 10-microsecond temporal resolution and at angstrom precision. Hence, they found correlations between the physical conformational dynamics of loop L6 probed by HS-AFM-HS and the kinetic details of voltage-dependent gating determined by single-channel electrical recordings on planar lipid bilayers (Figure 5). These explorations were supplemented by MD simulations, which provided additional atomic details of the coexistence of the open and closed states made by fluctuations of loop L6. These studies aimed at a better understanding of the gating activity of OmpG, stimulated by the prospects of using this monomeric β barrel in biosensing applications [128,131,137,138,139,140,141,142,143].

In the second example, Ferric hydroxamate uptake component A (FhuA), a monomeric 22-stranded β-barrel of *E. coli*, was extensively engineered for a better understanding of the gating activity produced by its large extracellular loops and the N-terminal 160-residue plug domain [144,145,146,147,148]. These studies, which involved its functional reconstitution into lipid bilayers, revealed the complexity of different contributions of the cork and loops to the gating activity of this TMP. The primary function of FhuA is to mediate the active high-affinity Fe^3+^ uptake into the cell [149]. A minimal 455-residue FhuA variant, which featured complete deletions of the plug domain and the large extracellular loops (L3, L4, L5, L10, L11), showed a quiet open-state conductance of ~1.6 nS in 300 KCl [150]. This relatively large single-channel conductance results from the passage of ions across an elliptical internal pore with sides of 2.6 × 3.6 nm. This FhuA variant was frequently employed for further developments in biosensing applications because of its monomeric nature, high thermodynamic stability, and relatively larger size [150,151,152,153,154,155,156].

Another example of a well-studied plug-containing OM protein is that of the usher pyelonephritis-associated pili C (PapC) [18,157,158]. This β barrel is a key element utilized by Gram-negative pathogenic bacteria (e.g., uropathogenic *E. coli*) to produce and assemble extracellular pilous fibers. PapC is a large 24-stranded dimeric, twin β-barrel complex with each monomer containing five functional domains: a β-barrel translocation domain, a β-sandwich plug domain, an N-terminal periplasmic domain, and two C-terminal periplasmic domains [18]. The wild-type PapC is mainly closed due to the β-sandwich plug domain [159]. However, this closed state is accompanied by short-lived openings to various substates. Further, the opening probability of PapC was increased by subsequent deletions of the N- and C-terminal domains, suggesting that they participate in the functional gating activity. Yet, the deletion of the plug domain resulted in extremely large single-channel conductance openings of the pore of ~3 and 7.3 nS for the monomer and dimer in 1 M KCl, respectively. Frequent closures decorated these open substates. This finding is in accordance with the measured internal size of 4.5 × 2.5 nm for the plug-deleted PapC monomer. Later, antibiotic sensitivity and electrophysiology measurements were employed to determine that a single salt was required to stabilize the 76-residue plug domain within the pore lumen [160]. In addition, it was identified that the loop between strands β12 and β13 mediates the pore opening.

**Figure 6:**
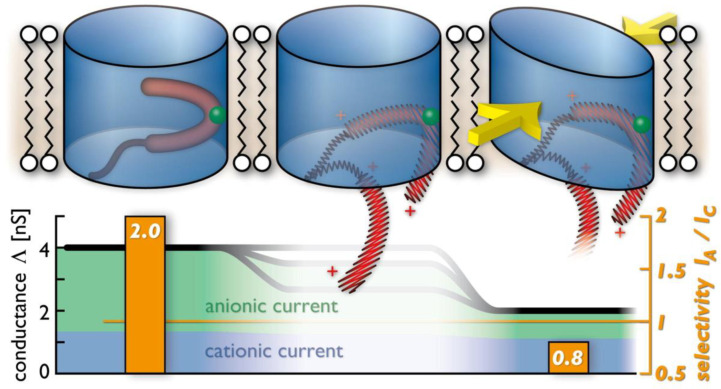
A proposed model for voltage sensing of VDAC1. VDAC1 (in blue) remains in a 4 nS-conductance open state at a zero transmembrane potential (upper, left). Yet, at an amplified applied transmembrane potential greater than 30 mV, regardless of its polarity, an electric force is exerted on the N-terminal helix that acts as a voltage sensor (in red; center). L10 (in green) is the contact residue of the N-terminal helix with the V143 residue on the barrel wall. The reversible dissociation of the rigid N-terminal helix from the pore wall results in a more flexible structure, which is likely to switch the channel into a semi-collapsed, elliptical conformation that leads to a 2 nS conductance closed state (upper, right). The lower panel indicates the correlated values in the open and closed state unitary conductance and ionic selectivity. This figure was adapted from Zachariae and coworkers (2013) [136].

**Figure 7:**
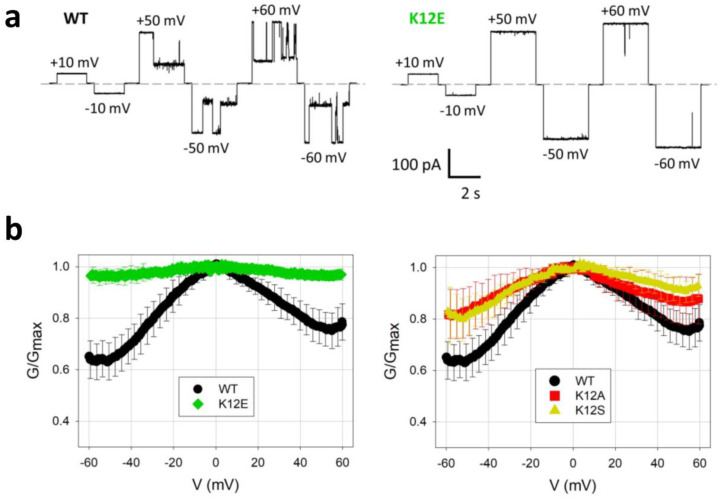
Direct experimental evidence for the implication of a key charged residue in the voltage-dependent gating of VDAC1. (**a**) Single-channel electrical recordings of mVDAC1 reveal the intense gating activity of the wild-type channel (left traces) but the drastically declined gating activity of the charge-reversal K12E mutant (right traces). Horizontal dashed lines show the zero current. (**b**) These panels indicate quantitative assessments of the gating activity of different VDAC1 proteins using a multichannel system. The vertical axis indicates the overall multichannel current normalized to the value corresponding to open-state multichannel conductance. The left panel compares the wild-type (WT) protein and the charge-reversal K12E mutant. The right panel compares the WT protein as well as the K12A and K12S mutants. This figure was adapted from Ngo and coworkers (2022) [161].

Below, Figure 6 and Figure 7 are presented.

## 4. Gating Activity Modulated by the N-Terminal Tail

Among mitochondrial OM proteins, the VDAC protein has drawn significant interest because of its critical regulatory implications in the metabolic operation of mitochondria under physiological and pathological conditions. The primary role of this multitasking β-barrel protein is to facilitate the exchange of ions, nucleotides, and metabolites between the mitochondrion and the cytosol [43,61,162,163,164,165,166,167]. Yet, its functional characteristics extend to that of a receptor for small molecules and proteins that regulate the overall OM permeability of mitochondria [165,167,168,169,170,171]. Human VDAC isoform 1 (hVDAC1) is the most abundant protein in mitochondrial membranes. The fundamental and translational implications of hVDAC1 in cell physiology and disease development have ignited numerous structural [63,135,172,173,174,175,176], biophysical [64,65,177,178,179,180,181,182,183,184], and functional [61,162,163,164,165,167,170,185,186,187,188,189,190,191] studies. Because of its pivotal role in interactions with apoptotic and anti-apoptotic proteins [165,168,169,190], hVDAC1 can potentially serve as a therapeutic target in diverse diseases, including several cancers, as well as cardiac and neurodegenerative pathologies [66,182,192,193,194,195,196].

The three-dimensional structure of VDAC1 has illuminated a 19-stranded β barrel with a partly α-helical N-terminal segment protruding into the pore lumen [60,173,197,198,199]. It is also worth mentioning that the first and last strands orient parallelly, making VDAC1 part of a unique subclass of β-barrel proteins. The channel exhibits a 4.1 nS conductance at transmembrane potentials lower than 30 mV and in 1 M KCl [200]. At elevated voltages greater than 30 mV, VDAC1 switches into a low-conductance closed state of ~2 nS. This process is symmetrical with respect to the polarity of the applied transmembrane potential. The transition from the large-conductance open state to the low-conductance close state also involves a drastic change in the ionic permeability from an anion- to a cation-selective pore [61,166,201]. Hence, it was postulated that extensive conformational changes of the N-terminal α-helix domain of the channel are responsible for its voltage-dependent alterations in the unitary conductance and ionic permeability. This interesting hypothesis stimulated further explorations of voltage sensing of this mitochondrial channel. To address this fundamental gap, Tejido and coworkers (2012) [202] engineered a double-cysteine mutant in murine VDAC1 (mVDAC1) to lock the N-terminal helix to the barrel wall. This was accomplished through L10C and A170C mutations on the N-terminal α helix and β strand 11, respectively, fixing the α helix to the pore wall. Surprisingly, the functional reconstitution of this mVDAC1 mutant, which encompasses a pore-lining N-terminal helix, did not reveal significant changes in the voltage-gating activity. This outcome suggested that the N-terminal helix remains linked to the pore wall while transitioning from the open to the closed state.

In an independently conducted study, Zachariae and colleagues (2012) [136] utilized electrophysiology, MD simulations, and solid-state NMR spectroscopy to reveal that the absence of the N-terminal helix enhances the breathing conformational fluctuations of the barrel wall. Therefore, deleting the N-terminal helix of hVDAC1 catalyzes the transition of its open-state conformation to a partly collapsed, closed-state conformation. The rigid N-terminal helix, which is deeply located within the pore lumen, stabilizes the channel in the high-conductance state. In addition, they found that a transient dissociation of the N-terminal helix from the barrel wall is a mechanism for switching the channel into a partially collapsed state, explaining both the unitary conductance and ionic selectivity of the closed state (Figure 6).

Voltage-dependent gating activity of VDAC1 is also modulated by other environmental or physical factors, such as lateral membrane pressure [100,189], pH [203], and temperature [204]. Recently, substantial progress has been made in a better mechanistic understanding of the most sensitive site involved in the voltage-dependent gating activity of VDAC1. Noskov and colleagues (2022) employed full atomistic MD simulations, X-ray crystallography, and electrophysiology to determine that the K12 residue in the N-terminal helix is a focal point of the voltage gating of mVDAC1 [161]. This study revealed coordinated motions of internal charged residues with conformational alterations in the cross-sectional β-barrel geometry. K12 fluctuates between two distinct energetic substate minima. Its motions between the two substates amplify the barrel fluctuations, leading to channel gating. Remarkably, the K12E mutant exhibited a structure like that of the wild-type (WT) protein, yet with a restricted motion of E12 residue due to its interactions with adjacent side chains. In accordance with structural data, MD simulations suggested that this single-residue K12E substitution resulted in the stiffening of the channel wall, causing low-amplitude conformational fluctuations of the barrel. These fluctuations diminished barrel motions of the K12E mutant and prevented channel gating (Figure 7a). Further, multichannel electrophysiology experiments showed that the single-site mutations of K12 to alanine, serine, or glutamine significantly declined the gating activity of mVDAC1 (Figure 7b).

## 5. Modulation of the Voltage-Dependent Gating by Environmental Conditions

### 5.1. Effect of pH

The impact of acidification on the gating activity has been observed by various groups using different β-barrel proteins, such as staphylococcal α-hemolysin [205,206,207,208], OmpC [87], OmpF [97,209,210], OmpG [116,117,129,133], and VDAC [203,210,211]. For example, the single-channel electrical signatures can span a wide range of gating fluctuations at an acidic pH, from an enhancement in the current noise [205,206] to large-amplitude current closures [207,208]. The acidification at pH values lower than the p*K*_a_ (e.g., ~4.0, which is comparable to those of titrable Asp and Glu residues) potentially destabilizes stiffer barrel regions due to the disruption of salt bridges. If titrable salt bridges are in the voltage sensing domain of the pore, then their perturbation likely accelerates the switching of the pore from the open to the closed state. A systematical analysis of the pH dependence of the gating of mVDAC1 revealed an asymmetric effect of the acidification with a prominent effect on the cytosolic side and a modest impact on the mitochondrial intermembrane side [203]. The numerous stable salt bridges at the cytosolic side of mVDAC1 caused this asymmetric effect. Moreover, the acidification enhanced the single-channel conductance and anion selectivity because of the titrable negatively charged residues at very low pH values.

Prior studies of the pH dependence of the voltage-dependent gating of OmpG [116,117,129,133] revealed loop L6 partitions into the pore lumen under acidic conditions, thus gating this monomeric porin. Min Chen and colleagues (2018) [212] determined that two charged patches within the pore lumen of OmpG are responsible for the increased gating activity of this OM protein at acidic pH values. Using a computational approach and single-molecule electrophysiology, they discovered that electrostatic interactions formed between loop L6 and charged residues on the barrel wall could be attractive or repulsive at a specified pH value. This way, a new strategy was developed for shifting the gating equilibrium by balanced protonation and deprotonation of essential histidine, aspartate, and glutamate side chains on the pore wall and loop L6. Recently, this approach inspired further engineering of OmpG for improved sensing capabilities at acidic pH [213]. Therefore, substituting charged residues on loop L6 with neutral side chains generated a relatively stable OmpG variant under a wider pH range. These redesign efforts show promise for developing and validating novel engineered OmpG nanopores for medical biotechnology.

### 5.2. Effect of Temperature

**Figure 8:**
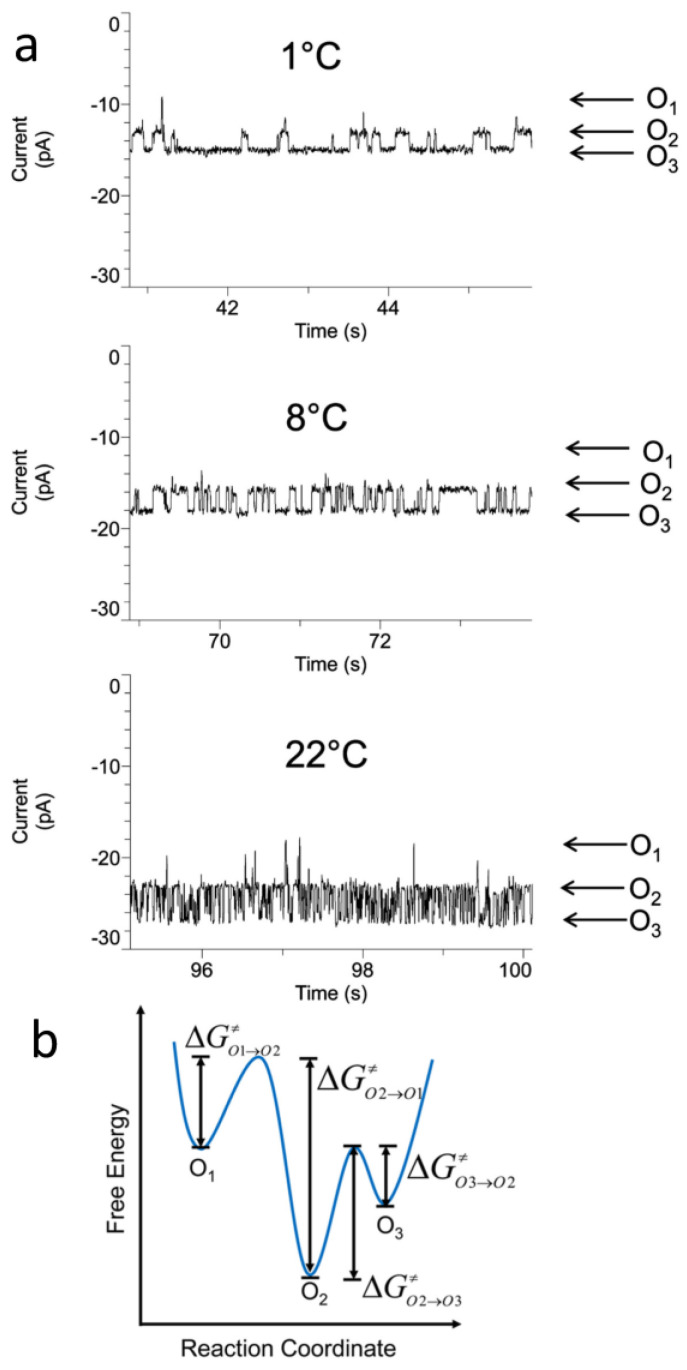
Temperature dependence of conductance substates of OpdK. (**a**) Single-channel electrical traces collected with the native OpdK at various temperatures. (**b**) A free energy landscape model illustrating the kinetic transitions among the O_1_, O_2_, and O_3_ open substates. This model shows the activation free energies characterizing various kinetic transitions (Δ*G*_O1→O2_^‡^, Δ*G*_O2→O1_^‡^, Δ*G*_O2→O3_^‡^, and Δ*G*_O3→O2_^‡^). This figure was adapted from Cheneke and coworkers (2015) [221].

A better understanding of the nature of conformational transitions of β barrels can be acquired by temperature dependence experiments. These studies illuminate thermostability features [214] of OM proteins when functionally reconstituted into lipid membranes, vesicles, or nanodiscs [215,216]. It should be mentioned that significant temperature alterations substantially affect various factors such as the ionic and sub-molecular diffusional mobilities, solvation layer, and unitary conductance. MD simulations and single-molecule electrophysiology provide information on the mechanisms of temperature-dependent changes in the unitary conductance of β-barrel proteins [217,218,219]. The difference between the unitary conductance of the barrel and its value corresponding to temperature-dependent solution conductivity is primarily accounted for by the significant interactions between translocating ions and surface charges on the pore wall. Electrophysiological measurements under different temperature conditions may potentially identify new open and/or closed substates where the barrel resides with the highest probability. For example, OmpA of *E. coli* undergoes interconvertible open states between small-conductance channels (e.g., 36–140 pS in 1 M KCl), between 15 and 37 °C, and large-conductance channels (e.g., 115–373 pS), between 15 and 37 °C [220]. At elevated temperatures, the ratio of the numbers of small- and large-conductance channels was altered, illustrating the dynamic coexistence of differently refolded barrels. A β barrel can also undergo significant changes in the equilibrium dynamics between its different substates. This situation occurs when the channel exhibits multiple substates resulting from complex interactions of different regions of the barrel. For example, OpdK of *P. aeruginosa* [37] shows three open substates, O_1_, O_2_, and O_3_ [123]. At room temperature, the most probable substate is O_2_ (Figure 8a). However, O_3_ is the most probable substate at 4 °C. Temperature changes can reveal modifications in the activation free energy barriers required to transition from one substate to another (Figure 8b) [221]. The primary benefit of single-molecule electrophysiology is the ability to determine the average time constants corresponding to individual substates [222]. This way, the precise nature of the gating mechanism is uniquely determined by obtaining the enthalpic and entropic contributions to the kinetic and thermodynamic constants, revealing which process in the gating transition is dominant [221,223,224,225,226,227]. For example, these analyses reveal enthalpy- and entropy-driven conformational transitions [204,227]. In addition, they provide quantitative assessments of extensive entropic changes in the gating transitions that are compensated by large enthalpic alterations in the form of enthalpy–entropy compensation [221,228,229]. Finally, temperature scanning of β-barrel proteins also has practical importance for identifying their thermostability in applications of biosensor technologies [208,225,226,230].

Below, Figure 8 is presented.

### 5.3. Effect of Lipid Composition and Bilayer Asymmetry

As part of the surrounding environment, the membrane’s composition may play a regulatory role in the voltage-dependent gating of a β-barrel protein through direct lipid–protein interactions [231,232]. Hence, lipid composition is an additional controlling factor, given its modulatory influence on mitochondrial and bacterial homeostasis. If both membrane monolayers consist of lipids with a relatively short spontaneous curvature, also called lamellar lipids [233], the elastic pressure within the hydrophobic core is modest. In this case, the lipid environment does not typically impact the gating dynamics of the channel because of its robust barrel scaffold. Yet, the presence of inverted hexagonal phase-forming lipids, also called nonlamellar lipids [234,235,236], exerts a substantial lateral packing pressure within the hydrophobic bilayer region, catalyzing ample thickness fluctuations of the membrane. Therefore, nonlamellar lipids significantly impact the gating dynamics of β barrels, despite their apparent mechanical robustness [237]. For example, phosphatidylethanolamine (PE) and cardiolipin (CL), two dominant lipids in the OMs of mitochondria, amplify the VDAC gating at negative applied potentials due to their high packing pressure [100]. Queralt-Martin and colleagues (2019) [189] systematically examined the influential role of the polar headgroup of membrane lipids on the voltage gating of mVDAC1. The charge of the phospholipid headgroup has a substantial effect on the channel gating as well. The positive charge of the headgroup amplifies gating, whereas the negative has a suppressing effect. This outcome reinforces the critical importance of the interfacial electrostatic forces between adjacent lipids and the membrane-solvated side of the mVDAC1 channel. Moreover, the same study clarified that the E73 residue, which faces the hydrophobic side of the channel, is not directly involved in the gating mechanism.

Further, the interfacial electrostatic forces at the protein–lipid interface may also have a dominant role. The high local densities of acidic and basic side chains cluster on the hydrophobic side of the channel and in the proximity of the polar headgroup region of lipids, such as in the case of OmpF [238]. Because of their titrable nature, these local charge densities make the –lipidprotein binding mechanism pH sensitive. Therefore, further explorations are needed for a better understanding of the effect of lipid charges on critical functional aspects of Gram-negative bacteria, such as the antibiotic uptake through porin-facilitated routes. However, there is recent experimental evidence that the hydrocarbon tails of lipids also play an essential task in modulating the voltage-dependent gating of OmpF [239]. Such an influential role of the hydrophobic core is likely achieved through the reorganization of the OmpF trimer by adopting different local conformations of individual monomers.

OMs of Gram-negative bacteria, mitochondria, and chloroplasts have an asymmetric distribution of lipid species in both leaflets. Hence, changes in the asymmetric composition, relative distribution, and physicochemical properties of lipid constituents across these membranes may impact the voltage-dependent gating dynamics of their β-barrel TMPs. Hwang and coworkers (2008) [240] used a droplet interface bilayer to produce the functional reconstitution of OmpG of *E. coli* into an asymmetric lipid bilayer with a positively charged monolayer opposing a negatively charged monolayer. Interestingly, they identified different gating signatures of OmpG that depended on the insertion leaflet of the asymmetric bilayer.

## 6. Applications in Biotechnology

This review article is focused on the biophysical mechanisms of voltage gating of β-barrel proteins. As mentioned above, many of these studies have been stimulated by prospects of employing these protein scaffolds in applied areas of biosensing and medical biotechnologies. The structural integrity and high thermodynamic stability of β barrels make them robust and versatile nanostructures, while the current modulation produced by their interaction with other molecules provides a sensitive readout [126]. These two properties create opportunities to develop powerful single-molecule sensors for various applications in molecular biomedical diagnostics and environmental monitoring [127,241,242,243]. For example, β barrels are utilized in DNA sequencing [244]. In addition, they are employed in the detection [245,246,247], chemical modification [248], and sequencing [249,250] of proteins. Specifically, MspA [251] and α-hemolysin [252] are examples of β barrels optimized for DNA sequencing, while proteins like FhuA [208] and OmpG [131] have been engineered to detect numerous target proteins. As basic research rapidly progresses, more β-barrel proteins are redesigned to address persistent demands and technical shortcomings in nanobiotechnology.

## 7. Concluding Remarks

In this review article, we briefly recapitulate elements concerning the structure and composition of these β-barrel protein pores, porins, and channels. The primary aim is to critically discuss the mechanisms of intrinsic voltage-dependent gating of these TMPs. Further protein engineering of barrel proteins will likely generate novel redesigned scaffolds for medical biotechnology. Moreover, an enormous body of literature concerns voltage gating of VDAC1 due to its regulatory mechanisms in mitochondria under physiological and pathological conditions. Several voltage-gating issues remain unresolved, so more developments and efforts are necessitated for their comprehensive and quantitative understanding. These fundamental gaps will likely be addressed in the future by utilizing high-resolution technologies both in a cell-free environment and in living cells.

## Data Availability

Not applicable.
